# A rare case of late-onset amyloidosis cutis dyschromica

**DOI:** 10.1016/j.jdcr.2024.05.037

**Published:** 2024-06-14

**Authors:** Megan Lau, Ata Moshiri, Shadi Khalil, Louis Siegel

**Affiliations:** a The Ronald O. Perelman Department of Dermatology, New York University Grossman School of Medicine, New York, New York; b New York University Grossman Long Island School of Medicine, Mineola, New York

**Keywords:** amyloidosis, amyloidosis cutis dyschromica, dermatomyositis, poikilodermas, dyschromatoses

## Introduction

Amyloidosis cutis dyschromica (ACD) is a rare type of primary cutaneous amyloidosis characterized by various patterns of widespread dyschromia, minimal pruritus, prepubertal onset, and amyloid deposition in the subepidermis.^1^ There are fewer than 40 reported cases since its first description in 1970.^2^ The familial disorder has predominantly been documented in Japan and China, with a single case reported in Mexico and a few instances in India.^3^ Genetic factors and impaired DNA repair from UV light are suspected contributors to the etiology of ACD.^4^ Different treatment modalities, including sunscreen, topical corticosteroids, keratolytics, dimethyl sulfoxide, capsaicin, CO2 laser, and acitretin, have been used with varying degrees of effectiveness.^4^ Here, we describe a rare presentation of ACD demonstrating classic histopathologic features, but notably with late onset, localized presentation, and no familial history.

## Case report

**Fig 1:**
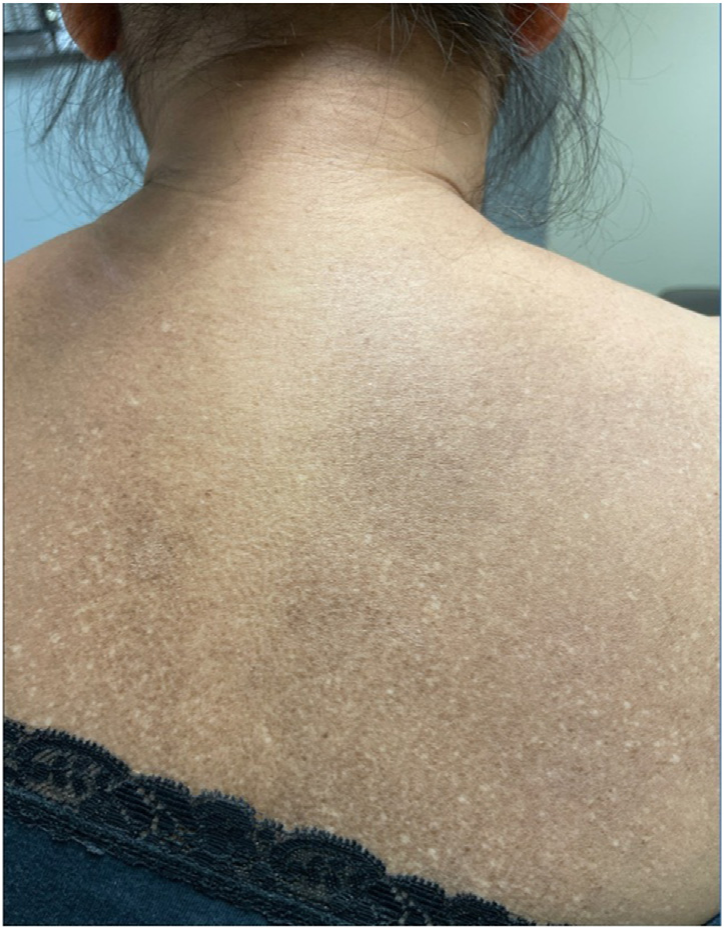
Hypopigmented macules with hyperpigmented macules and patches on upper back.

A 52-year-old woman presented to the outpatient dermatology clinic with an 8-year history of hypopigmented macules interspersed with hyperpigmented macules and patches on the back (Fig 1). There were no lesions present on the extremities or anterior trunk. Her past medical history was significant for diabetes mellitus type 2, hyperlipidemia, and lichen simplex chronicus on the right lower extremity controlled with tacrolimus 0.1% ointment. The patient denied any family history of dermatologic conditions, photosensitivity, blistering, itching, erythema, dysphagia, or muscle weakness.

**Fig 2:**
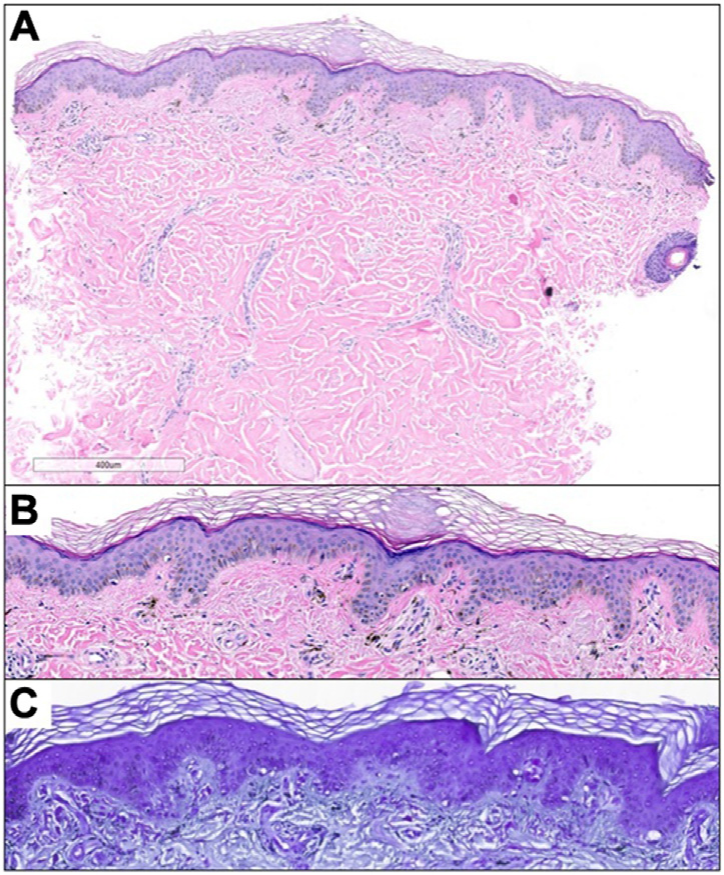
Representative histopathology. **A** and **B,** Punch biopsy from the skin demonstrated deposition of amorphous material and abundant melanophages in the papillary dermis by hematoxylin and eosin staining. **C,** Positive labeling of amyloid was observed following special stain with crystal violet.

A 2 mm punch biopsy from the right superior back revealed amorphous material and a significant accumulation of melanophages within the papillary dermis on hematoxylin and eosin sections (Fig 2, *A* and *B*). Subsequent crystal violet stain confirmed the presence of amyloid (Fig 2, *C*). Taking into account the clinical presentation, a diagnosis of ACD was rendered. The patient was educated about the benign nature of the condition, and recommendations for sun avoidance and protection were provided.

## Discussion

This case differs from other reports in the literature due to the late onset in adulthood, localized rather than diffuse presentation, and lack of family history.^3^^,^^4^ Reports of postpubertal onset cases of ACD are very rare, with only one known case to our knowledge involving a 49-year-old woman with widespread reticular and speckled pigmented lesions.^4^ In a single-center retrospective review of 10 cases of ACD, half were from the same family (*n* = 5), with most cases occurring in childhood (*n* = 7). The condition was characterized by mottled hyperpigmented and hypopigmented macules accompanied by mild itching or no symptoms. There were no reports of systemic amyloidoses, and amyloid deposits were present in the papillary dermis and tested positive with Congo red stain.^2^ A similar asymptomatic presentation with extensive reticulate hyperpigmentation and hypopigmentation was observed in another case report of 2 male siblings, aged 25 and 20, and in another case report of a 26-year-old female with lesions present since birth.^3^^,^^5^

Our patient's histologic findings align with the classic histopathology of ACD.^5^ Of note, macular amyloidosis has similar histologic findings, emphasizing the importance of clinical correlation. Histologic features of macular amyloidosis often include amorphous eosinophilic deposits in the papillary dermis, a characteristic also observed in ACD.^6^ Additionally, positive immunofluorescence for amyloid in the papillary dermis is a shared feature between the 2 conditions. The main distinguishing factor lies in their clinical presentation. Macular amyloidosis typically appears as rippled hyperpigmented macules in areas of constant friction, while ACD occurs more commonly in sun-exposed areas with mottled hyperpigmented and hypopigmented lesions.^6^

Dyschromatoses including the 2 major forms dyschromatosis symmetrica hereditaria and dyschromatosis universalis hereditaria, poikilodermas, and dermatomyositis, were considered in the differential diagnosis. Critical distinguishing features are summarized in Table I. While all conditions may be asymptomatic, poikilodermas are often associated with burning and itching and dermatomyositis may also present with other cutaneous findings (ie, heliotrope rash, Gottron’s papules, and alopecia), pruritus, and nondermatologic features such as symmetric and proximal muscle weakness.^7^, ^8^, ^9^

**Table I:** Differential diagnosis of ACD

	ACD	Dyschromatoses (dyschromatosis symmetrica hereditaria dyschromatosis universalis hereditarian)	Poikilodermas	Dermatomyositis
Etiology	Amyloid deposition in the skin	Autosomal dominant inheritance of impaired melanin synthesis and melanosome sorting	Chronic sun exposure	Idiopathic, autoimmune, or paraneoplastic associated with non-Hodgkin lymphoma, lung, stomach, colorectal, or ovarian cancer
Dermatologic clinical presentation	Reticular hyperpigmented or hypopigmented macules with diffuse distribution	Hypopigmented and hyperpigmented lesions approximately 5 mm on hands and feet and freckles on face	Hyperpigmented or hypopigmented lesions, telangiectasias, and atrophy in sun exposed areas.	Erythematous rash located joints on extensor hand surface (Gottron sign), upper eyelids (heliotrope rash), superior anterior trunk (V-sign), and inferior posterior trunk (shawl sign)
Age of onset	Prepubertal	Infancy or Early childhood	Adult and rarely early childhood	Juvenile and adult
Associated symptoms	Asymptomatic or minimal signs of pruritis	Asymptomatic	Asymptomatic or skin tightness, pruritus, burning sensation	Pruritus, symmetric and proximal muscle weakness, panniculitis, alopecia
Histopathologic findings	Subepidermal amorphous eosinophils (amyloid) deposition, hyperkeratosis, melanophages	Increase or decrease in basal layer’s melanin and pigmentary incontinence	Atrophy, telangiectasias, hyperkeratosis and lymphohistiocytic infiltration in the dermis and epidermis.	Chronic nonspecific or interface dermatitis, atrophy with vacuolar interface changes, perivascular lymphocytic infiltrate with increased mucin

*ACD*, Amyloidosis cutis dyschromica.

The histopathologic findings are the primary distinguishing factor among these conditions, which may share similar clinical presentations. ACD presents with amyloid deposition and melanophages within the papillary dermis, while dyschromatoses may present with focal changes in the basal layer's melanin content and pigmentary incontinence.^10^ Poikiloderma’s histologic findings include lymphohistiocytic infiltrates in the dermis and epidermis, hyperkeratosis, telangiectasia, and solar elastosis.^7^ Further, the histologic findings of dermatomyositis present similar to systemic lupus erythematosus with chronic nonspecific or interface dermatitis, atrophy with vacuolar interface changes, and light perivascular lymphocytic infiltrate with increased mucin.^9^^,^^11^ Although the typical prepubertal onset of ACD may be helpful in its differentiation from dyschromatoses and poikilodermas, our case highlights that this feature may not be reliably distinctive, as ACD can manifest later in life. In summary, our case demonstrates the importance of skin biopsy and histopathologic findings in the definitive diagnosis of ACD.

## Conflict of interest

None disclosed.
